# Extrachromosomally co-encoded toxin-immunity pairs in yeast and their application potential

**DOI:** 10.1007/s00253-026-13876-0

**Published:** 2026-05-26

**Authors:** Thijs de Vroet, Rianne C. Prins, Sonja Billerbeck

**Affiliations:** 1 Molecular Microbiology, Groningen Biomolecular Sciences and Biotechnology Institute, University of Groningen, Groningen, 9747 AG The Netherlands; 2 Department of Bioengineering, Imperial College London, White City Campus, Translation and Innovation Hub (iHUB), London, W12 0BZ UK

**Keywords:** Yeast, Toxins, Immunity, Extrachromosomal elements, Synthetic biology

## Abstract

**Abstract:**

Some yeast isolates secrete toxic proteins called yeast killer toxins, which kill other yeast and filamentous fungi through toxin-specific modes of action. These toxins hold potential as food preservatives, as therapeutics, and for biotechnology. Toxin production typically necessitates self-protection during and after secretion, as several toxins also act against their own producer species. Self-protection can occur through genomic mutations or through dedicated immunity factors. Here, we focus on dedicated immunity factors that are encoded on the same extrachromosomal element as their cognate toxin. These co-encoded toxin-immunity pairs reside on cytoplasmic elements that rely on autonomous replication for their maintenance and expression: either as double-stranded RNA (dsRNA) mycovirus satellites or double-stranded DNA virus-like elements (VLEs). On these systems, the immunity factors are either encoded within the same open reading frame (ORF) as their cognate toxin or encoded on a separate neighboring ORF. While the mode of action of several toxins has been studied, relatively little is known about the mode of protection of their immunity factors. Here, we consolidate current molecular knowledge of exemplary toxin-immunity pairs and the methods used to clone, study, and engineer them, with the goal of advancing fundamental understanding and enabling their use in synthetic biology and biotechnology, analogous to intracellular bacterial toxin-antitoxin systems that have historically been used in synthetic biology.

**Key points:**

• *Yeast extrachromosomal elements encoding toxins often also encode immunity factors.*

• *Systematic studies have increased molecular understanding of toxin-immunity pairs.*

• *When understood further, toxin-immunity pairs could be used in synthetic biology.*

**Supplementary Information:**

The online version contains supplementary material available at 10.1007/s00253-026-13876-0.

## Introduction

Yeast killer toxins, also called mycocins (Valero et al. 2025), are ribosomally synthesized proteins that are lethal to yeast and filamentous fungi. These toxins are diverse in their species specificity, their activity in different environments and their modes of action. For some toxins, such as HM1, killing activity against various species in different environments has been reported (Lowes et al. 2000). For others, such as K1 and K2, killing activity appears limited to a few species at specific pH and temperature (Kurzweilová and Sigler 1993; Prins et al. 2026). 

These killer toxins are produced by so-called “killer yeasts” (Travers-Cook et al. 2023), and several reviews have compiled information on specific types of killer toxins (Mannazzu et al. 2019; Schaffrath et al. 2018), the discovery of new killer yeasts (Billerbeck et al. 2024; Boynton 2019; Maciá Valero et al. 2025), their potential applications (Mannazzu et al. 2019; Schaffrath et al. 2018), and how they fit into the broader landscape of antifungal compounds secreted by yeast (Maciá Valero et al. 2025).

Killer toxins can be encoded on the nuclear genome of a killer yeast, or they can be encoded on extrachromosomal elements that reside in the cytoplasm. These extrachromosomal elements include double-stranded RNA (dsRNA) satellites which are maintained by dsRNA mycoviruses (Crucitti et al. 2021; Rowley 2017; Vijayraghavan et al. 2023) and double-stranded DNA (dsDNA) virus-like elements (VLEs), previously named linear plasmids or killer plasmids (Klassen and Meinhardt 2007).

Frequently, killer toxins target their own producer species, and thus, killer-toxin-producing yeast isolates need to encode a mechanism to prevent self-killing. Such self-protection mechanisms have been termed immunity or resistance (Breinig et al. 2006; Finkler et al. 1992), with immunity becoming the more prominent term in the yeast killer toxin field (Creagh et al. 2025; Prins and Billerbeck 2024).

Noteworthy, toxicity, sensitivity, and immunity are dose and time-dependent phenomena. From an experimental standpoint, the killer toxin field has used growth inhibition observed in a halo assay as a measure for sensitivity. Using this phenotype shows limitations in resolution as it combines toxin production and sensitive strain fitness, but it has proven to be a useful assay for screening and has led to the discovery of most yeast killer toxins. Therefore, in this review, when we refer to sensitivity, increased tolerance or immunity, we refer to strain-specific observed growth inhibition, or absence thereof, in the presence of a killer toxin-producing strain or upon addition of toxin as reported in literature.

One way in which immunity in a toxin producer cell can be achieved is by genomic mutations. This is exemplified by the immunity to the dsRNA-satellite-encoded toxins KP6 (Finkler et al. 1992) and K62 (Creagh et al. 2025) which are produced by *Ustilago maydis* and *Saccharomyces paradoxus* isolates, respectively*.* Similar forms of genomic variations that lead to immunity or reduced sensitivity have been reported in non-producer cells. For example, *Saccharomyces cerevisiae* isolates with enhanced expression levels of the *PAU* genes showed higher tolerance to the killer toxin K2, which is produced by mycovirus-infected *S. cerevisiae* isolates (Rivero et al. 2015); Further, it was shown that alleles of the rapidly evolving ORF encoding *KTD1* (a member of the DUP240 gene family) provided increased tolerance to the K28 toxin in non-producer *S. cerevisiae* isolates (Andreev et al. 2023). Finally, deletion studies have revealed that loss of genes can result in increased or decreased sensitivity (Miyamoto et al. 2011; Pagé et al. 2003).

**Fig. 1:**
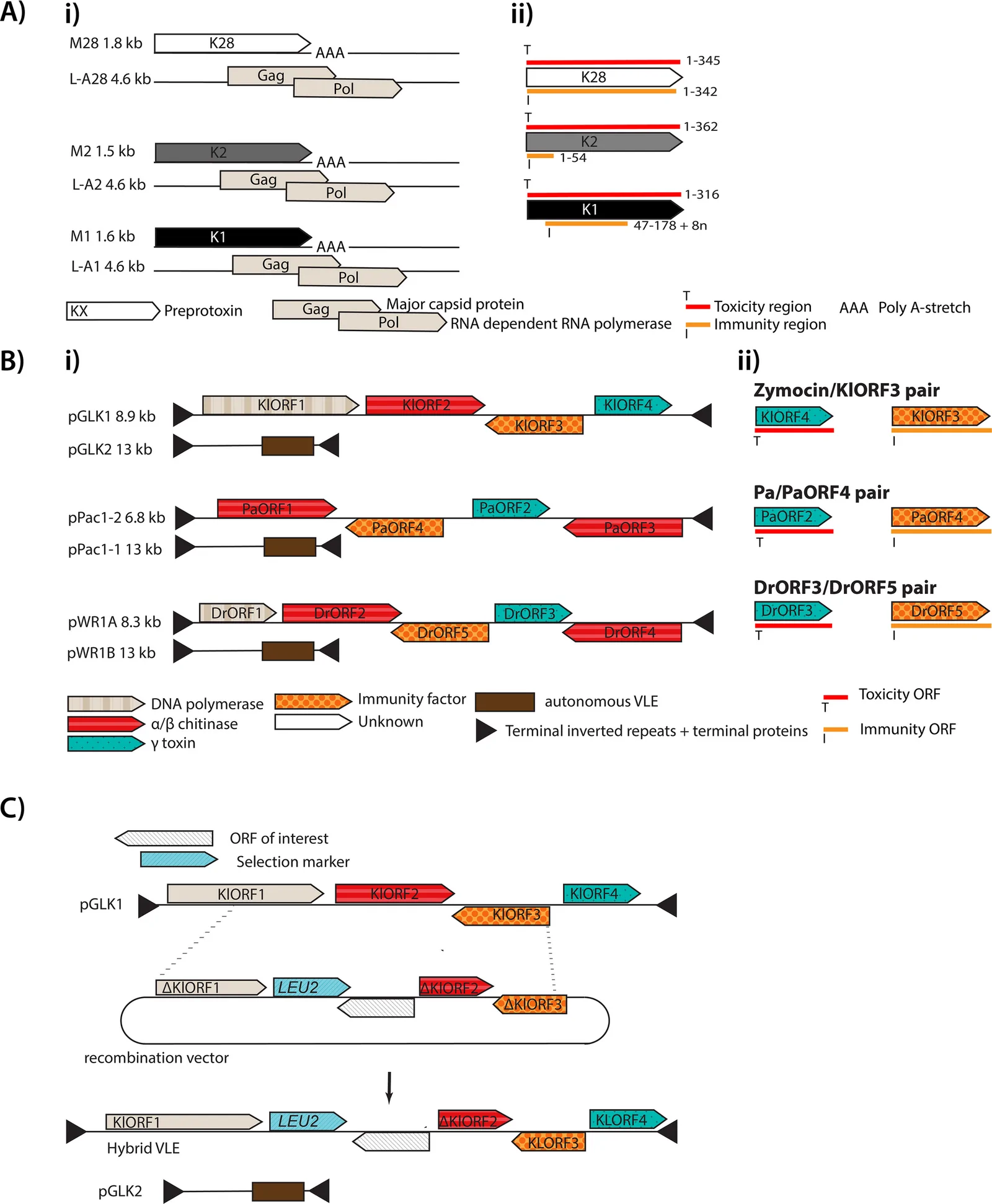
Extrachromosomal elements encoding toxin-immunity pairs. **A** (i) dsRNA satellites (M) encode a preprotoxin ORF followed by a poly-A region and non-coding region. Each dsRNA satellite relies on a specific L-A helper virus for transcription and packaging. L-A helper viruses encode the capsid protein Gag and an RNA-dependent polymerase Pol, which are used for self-replication and are capable of replicating and packaging dsRNA satellites. (ii) The preprotoxin sequence is sufficient for immunity and toxicity with different regions encoding both phenotypes, yielding single-ORF toxin-immunity pairs. **B** (i) Linear plasmids, also named VLEs, encode several ORFs responsible for immunity and toxicity. The mature VLE toxin is formed by an α/β-chitinase and the γ toxin. Each toxin-encoding VLE relies on the machinery of an autonomous VLE capable of replication, transcription, and transcript processing. Each VLE is flanked by terminal inverted repeats and the terminal repeats have bound proteins required for its replication. (ii) Each γ toxin is accompanied by a dedicated immunity factor encoded on the same VLE. **C** Using homologous recombination, an ORF of interest can be expressed on a hybrid VLE. First, a recombination vector carrying a selection marker and the ORF of interest flanked by two homology arms is transferred into the cell. After recombination, the newly formed hybrid VLE utilizes the pGLK2 autonomous VLE for expression of the ORF of interest and the selection marker. Cells carrying the hybrid VLE are subsequently selected and studied.

Self-protection in toxin-producing yeast can also be mediated by dedicated immunity factors, which are proteins or protein fragments that provide immunity in an often toxin-specific manner. Currently, all experimentally validated examples of dedicated immunity factors were found co-encoded with their cognate toxin on an extrachromosomal element. In such arrangements, the immunity factor is either embedded within the same open reading frame (ORF) as the toxin or encoded in a separate ORF, located close to the toxin gene, yielding co-encoded toxin-immunity pairs (Fig. 1).

From an application-oriented viewpoint, we therefore focus our review on those extrachromosomal co-encoded toxin-immunity pairs: First, their physical linkage facilitates confident pairing of toxins with their immunity factors, which has facilitated (and will continue to facilitate) discovery, cloning, and heterologous expression in both experimental and sequencing-driven workflows. Moreover, co-localization on an extrachromosomal element may have driven tight co-evolution, potentially yielding toxin-immunity pairs that are highly specific to one another and functionally orthogonal to host functions. Indeed, preliminary evidence supports high specificity between toxin-immunity pairs (Klassen et al. 2014), and cells are viable after the removal of the extrachromosomal element, indicating their dispensability and orthogonal function (Fredericks et al. 2021; Klassen and Meinhardt 2002; Połomska et al. 2021; Vijayraghavan et al. 2023). These properties offer potential for modular deployment of these systems in synthetic biology.

Examples of single-ORF toxin-immunity pairs, where expression of the ORF itself is necessary and sufficient for toxicity and immunity, are the toxins K1, K2, and K28, which are produced by *S. cerevisiae* isolates infected with the dsRNA satellites M1 (Russell et al. 1997), M2 (Dignard et al. 1991), or M28 (Schmitt 1995) and their respective dsRNA L-A-helper virus (Dignard et al. 1991; Russell et al. 1997; Schmitt 1995) (Fig. 1A). Examples of separate-ORF toxin-immunity pairs are the VLE-encoded toxins PaT (*Pichia acacia)*, zymocin (Kluyveromyces* lactis)*, DrT (*Debaromyces robertsiae)* and their respective immunity factors called after the ORFs that encode these factors, PaORF4 (Paluszynski et al. 2007), KlORF3 (Stark 1988; Tokunaga et al. 1987b), and DrORF5 (Klassen et al. 2014), respectively (Fig. 1B). These dedicated immunity factors show a range of sizes: While the immunity factor encoded within the single-ORF K2 toxin-immunity pair is a 54-amino-acid peptide encoded within the secretion signal of the ORF (Prins and Billerbeck 2024), the immunity factors PaORF4, KlORF3, and DrORF5 are larger proteins of 363, 452, and 353 amino acids, respectively.

Such yeast toxin-immunity pairs, akin to bacterial toxin-antitoxin systems (Lin et al. 2023; Qiu et al. 2022), have potential as selectable markers, biocontainment tools, and modules for creating interdependent consortia for bioproduction, but greater molecular insight and systematic and modular engineering would be needed.

In addition, when toxins are deployed as future therapeutics or biocontrol agents, a detailed understanding of their cognate immunity mechanisms remain essential for safe and effective application design. In the case of the K2 system, a minimal genetic element can confer resistance; delineating its sequence space and mutational tolerance will help anticipate resistance trajectories in prospective targets and risks for these small resistance factors to appear de novo (Prins and Billerbeck 2025b). Further, ecological studies on immunity factors have increased our understanding of yeast population dynamics (Giometto et al. 2021; Travers-Cook et al. 2023; Vijayraghavan et al. 2023), maintenance of the killer phenotype, and the potential for horizontal gene transfer of immunity genes (Heneghan et al. 2024a, b). Mapping the mobility and prevalence of immunity determinants can inform resistance-risk forecasts and guide stewardship strategies.

**Fig. 2:**
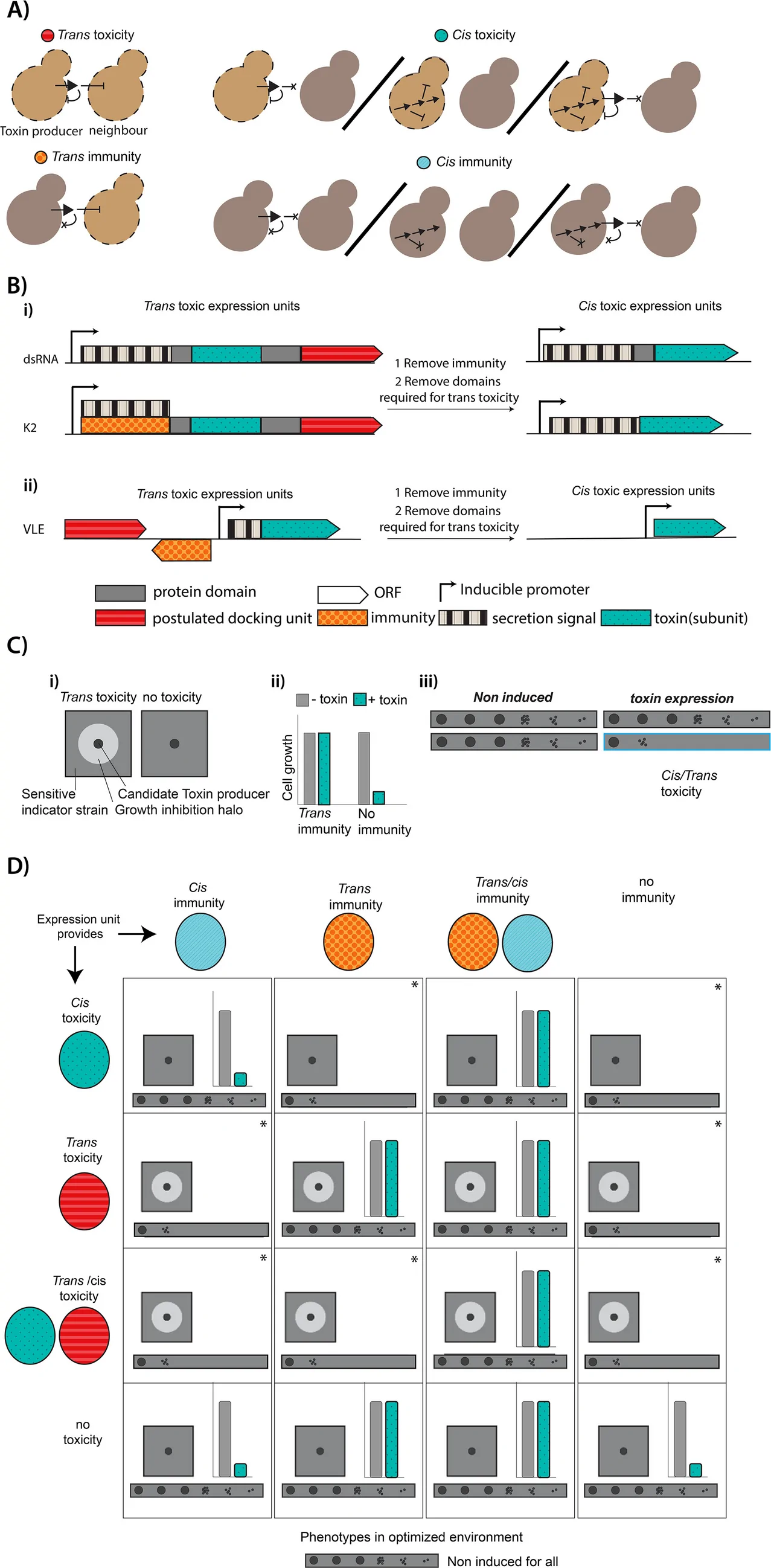
Concepts of and methods to study *cis* and *trans* toxicity and immunity phenotypes. **A**. A toxin’s activity can affect its producer and neighboring cells through several mechanisms, indicated here by solidi (/), resulting in two discernible phenotypes, given that an assay provides the right environmental conditions for the toxin to be active, e.g., pH and temperature. *Trans* toxicity is the ability of secreted proteins to inhibit the growth of sensitive yeast cells present in the same environment. *Trans* immunity is protection against these extracellular proteins. *Cis* toxicity is the growth-inhibitory effect of a toxin on its producer without affecting neighboring cells. *Cis* toxicity can occur intracellularly, with the toxin inhibiting producer growth prior to entering the extracellular environment. Alternatively, the toxin enters the extracellular environment but is unable to inhibit the growth of neighboring cells while inhibiting the growth of its producer. *Cis* immunity is the self-protection of a producer cell against the *cis*-toxic activity of the toxin it produces. **B** *Trans*-toxic expression units have shown to be convertible to display a *cis*-toxic phenotype through a series of steps, which are likely generalizable to other toxins: First, the toxic subunit is brought under the control of an inducible promoter. Second, known immunity factors are removed and third, domains required for *trans* toxicity such as cell-wall-binding domains, are removed. (i) Some dsRNA toxins require processing in the secretory pathway and therefore require a signal peptide. In the case of K2, the immunity/signal peptide needs to be substituted with a single-function signal peptide. Note that a simplified and abstracted version of K2 is shown; see Fig. 3A for a more detailed overview. (ii) The signal peptide of VLE toxins can be removed alongside the docking unit to construct a *cis*-toxic expression unit. **C** Through a combination of three separate assays, the different toxicity and immunity phenotypes can be studied. (i) *Trans* toxicity is observed using a halo-assay in which a (modified) toxin-producing strain is grown on an agar plate containing a sensitive indicator strain. (ii) *Trans* immunity is observed through extracellular addition of the wild-type toxin. In this assay, the growth of a strain expressing a genetic toxin construct in the presence or absence of a toxin is compared. iii) To gain insight into *cis*-toxic phenotypes, a spot assay is used. Dilutions of cells expressing a construct under the control of an inducible promoter, such as described under b, are spotted onto an inducing and non-inducing plate. **D** Using the outcome of the assays described under c, the toxicity phenotypes described under a can be systematically inferred. Scenarios labeled with a *indicate scenarios where the growth assay in the presence of externally added toxin can lead to various results, depending on the toxic activity of the investigated strain. Once deconvoluted, existing yeast toxin-immunity systems provide a growing toolbox for two use cases: (1) building toxin-antitoxin modules that act in *cis *on the producer cell and (2) secreted toxin systems that inhibit or kill neighboring cells in *trans*. Notably, until today, most *cis*-acting toxin-immunity variants have only been constructed for basic research, so they have not yet been systematically tested, standardized, or optimized for synthetic biology applications.

Here, we consolidate molecular knowledge on the six extrachromosomally co-encoded toxin-immunity pairs that have currently been experimentally validated and studied (i.e., those associated with the toxins K1, K2, K28, PaT, zymocin, and DrT). We first summarize the methods used to discover, clone, modify, and characterize these systems, including their ability to yield extracellularly and (engineered) intracellularly acting toxin-immunity pairs (Figs. 1C and 2). We then discuss specific examples of single-ORF and separate-ORF toxin-immunity pairs, including the available knowledge on their mechanisms of protection and orthogonality; followed by promising directions for future study and applications.

### Experimental and bioinformatic discovery, cloning, and heterologous expression of extrachromosomally co-encoded toxin-immunity pairs

Yeast with antifungal properties, potentially encoding killer toxins, are ubiquitous in the environment and have been isolated in various studies (Crabtree et al. 2023; Maciá Valero et al. 2025; Vijayraghavan et al. 2023). To gain insight into whether their killer phenotype is encoded on an extrachromosomal element or encoded on the nuclear genome, researchers have employed a “curing” approach in which potential extrachromosomal elements are removed (Fredericks et al. 2021; Vijayraghavan et al. 2023). Cured cells can then be tested for loss of toxicity, which is evidence that the toxin was encoded on a removed extrachromosomal element. dsRNA satellites can be cured by treatment with chemicals such as cycloheximide, anisomycin, or by high temperatures (Fredericks et al. 2021; Vijayraghavan et al. 2023), whereas UV light or saltless media cause loss of VLEs (Klassen and Meinhardt 2002; Połomska et al. 2021). Combined with assays to test for immunity and toxicity (Prins and Billerbeck 2025a) phenotypes, extrachromosomally co-encoded toxin-immunity pairs have been identified in several *Ascomycota* yeast (dsRNA satellites and VLEs) and *Basidiomycota* yeast (dsRNA virus) (see review for more details (Travers-Cook et al. 2023)).

The extrachromosomal elements can then be isolated by researchers from their non-cured hosts and sequenced. VLEs can be extracted through centrifugation of lysed host cells and sequenced by standard next-generation sequencing technologies (Połomska et al. 2021). Of note, VLE contigs are also readily recovered in whole-genome sequencing datasets, indicating that they co-purify with genomic DNA and do not require specialized isolation techniques (Heneghan et al. 2024a, b). dsRNA satellites can be isolated and sequenced via creating cDNAs (Crabtree et al. 2019).

Once sequenced, researchers started cloning the toxin-immunity-encoding ORFs to facilitate their study:

For many dsRNA-encoded toxins from *S. cerevisiae* mycovirus satellites, such as K1 (Gier et al. 2017), K2 (Meškauskas and Čitavičius 1992), and K28 (Schmitt 1995), the single toxin-immunity ORFs have been routinely cloned for decades into nuclear episomal high-copy plasmids (e.g., the pRS series) and expressed/secreted from laboratory strains of *S. cerevisiae* (e.g., BY4741). K28 has also been functionally secreted from other yeast, including *Schizosaccharomyces pombe* (Heintel et al. 2001) and *Komagatella phaffii* (formerly known as *Pichia pastoris*) (Giesselmann et al. 2017), while expression of K1 and K2 from heterologous yeast has not been published to the best of our knowledge.

Interestingly, for some VLE-encoded separate-ORF toxin-immunity pairs (PaT/PaORF4, zymocin/KlORF3, DrT/DrORF5, from *P. acacia*, *K. lactis*, and *D. robertsiae*, respectively), a nuclear expression barrier for the immunity ORF was discovered, which initially prevented their functional expression from conventional nuclear plasmids (Kast et al. 2015). This could however be overcome by recoding (Kast et al. 2015): The high A/T content (> 75%) of the immunity ORFs was shown to cause rapid mRNA fragmentation due to poly(A)-site processing. Consistently, lowering the A/T content prevented transcript cleavage and permitted functional nuclear expression (Supplementary Fig. 1).

Methods for direct genetic modification of the cytoplasmic VLEs have also been developed (Kamper 1989; Paluszynski et al. 2007; Tanguy-Rougeau et al. 1990) (Fig. 1C). One of the challenges in working directly with VLEs is that they cannot be modified by conventional in vitro manipulation, because of their special replication mechanisms working via protein-linked termini (Fig. 1B) (Paluszynski et al. 2007; Schickel 1996). Therefore, in vivo homologous recombination has been employed (Klassen et al. 2014; Larsen and Meinhardt 2000; Paluszynski et al. 2007; Schickel 1996), using linear dsDNA fragments with homology arms which flank a user-defined cargo and a cytoplasmic selection marker, driven by dedicated cytoplasmic promoters (VLEs have their own transcription machinery) (Fig. 1C). Those are typically used in the homologous recombination workhorse *S. cerevisiae*, also showing that VLEs can be transferred into other yeast hosts.

Expression from nuclear plasmids and/or from engineered hybrid VLEs eventually enabled researchers to confirm the function of several VLE-encoded toxin-immunity pairs such as PaT/PaORF4, zymocin/KlORF3, and DrT/DrORF5 **(**Fig. 1B). This paved the way for a recent systematic bioinformatic survey searching *Saccharomycotina* genomes for such pairs (Heneghan et al. 2024a, b), using genomic data curated to focus on VLE contigs which are usually omitted from genome assemblies. Here, zymocin was used as a query and 45 zymocin-like toxin candidates were identified, several of which were subsequently functionally validated by heterologous expression in *S. cerevisiae* (Heneghan et al. 2024a, b). These candidates were found to be encoded on VLEs but also within yeast nuclear genomes, the latter being a first indication that toxin-immunity pairs might not be restricted to extrachromosomal elements but also be found on the nuclear genome. Across genomic contexts, the toxin gene typically occurred adjacent to genes encoding a putative immunity factor, suggesting a conserved coding organization and thus substantiating the potential for bioinformatic discovery of additional pairs. Notably, in a second study, zymocin-like toxins and homologs of immunity proteins were also found in the genomes of filamentous fungi (*Pezizomycotina*) (Heneghan et al. 2024a, b).

bioinformatic searches for new extrachromosomal single-ORF toxin-immunity pairs are complicated by the fact that they are predominantly encoded on dsRNA viruses and their satellites, which are not assessed by genome sequencing efforts (Fredericks et al. 2021). However, several studies performed targeted dsRNA isolation and sequencing across *S. cerevisiae* isolates and other species within the *Saccharomyces* sensu stricto clade. This led to a better understanding of the prevalence of known toxins across *S. cerevisiae* isolates (Vijayraghavan et al. 2023), as well as the discovery of a new K1-like toxin (Fredericks et al. 2021) and a new toxin K62 (Rodríguez-Cousiño et al. 2017), which was later found to not be a single-ORF toxin-immunity pair instead relying on a currently unknown immunity mechanism (Creagh et al. 2025).

While it is still challenging to identify completely novel toxin-immunity systems from sequencing data alone, once a toxin-immunity pair has been validated it forms a starting point for sequence-based searches for more similar systems (e.g., as was done for zymocin). Recently, protein structure predictions have further revealed striking structural similarities among different yeast killer toxins, despite lacking a high level of sequence identity, supporting the existence of toxin superfamilies (Prins and Billerbeck 2024). As such, structure-guided searches using protein structure predictions may offer another approach to discover additional toxin-immunity systems.

### Concepts of *cis* and *trans* toxicity/immunity and methods to test for it

As we consider yeast toxin-immunity systems for synthetic biology, it is useful to highlight how they differ from those bacterial toxin-antitoxin systems that have historically been used in synthetic biology applications, both in bacteria (Lin et al. 2023; Qiu et al. 2022) and yeast (Duperray et al. 2023; Kristoffersen et al. 2000; Yeo et al. 2016). These bacterial systems are intracellular, where toxins harm only the producer, and antitoxins neutralize them inside the cell, often via tight binding (Prins and Billerbeck 2025). In contrast, natural yeast toxin-immunity pairs involve secreted toxins that harm neighboring cells and the producer, as such more akin to bacterial bacteriocin-immunity systems (Arbulu and Kjos 2024). Yeast toxins can also harm the producer during biogenesis, requiring protection of the producer both during intracellular production and against the mature extracellular toxin (Finkler et al. 1992). To differentiate between toxicity stages for the toxin producer, we propose utilizing the *cis/trans* toxicity distinction already used in the bacterial field for their secreted toxins (Oka et al. 2024) (Fig. 2A). *Cis* toxicity refers to the potential harmful effect of a toxin on its producer before being able to harm neighboring cells, and *trans* toxicity refers to a harmful effect after secretion or leakage of the mature toxin as it interacts with the producer and neighboring cells, also called fratricidal phenotype in other disciplines. *Cis* and *trans* immunity are consequently the mechanisms through which the producer cell protects itself against this toxicity. Here, it is however unknown if the same or different mechanisms apply for *cis* and *trans* immunity. Note that identical phenotypes can be the result of different molecular mechanisms (Fig. 2A).

So far, the only toxin for which both *cis* and *trans* immunity have been studied and postulated to be encoded by different stretches in the single-ORF is the toxin K1 (Zhu et al. 1993). For toxins that undergo retrograde transport into the cytosol and act intracellularly, such as PaT and K28, immunity is mediated by direct intracellular binding to a cognate immunity protein (Chakravarty et al. 2014) or the precursor (Breinig et al. 2006), accordingly, and the same immunity protein likely provides both *cis* and *trans* protection. For other toxins such as K2, it remains unknown if the cell is protected by one dedicated *cis/trans* immunity factor only, or if for *cis* immunity there are additional factors such as toxin inactivation until secretion by pH or by the proregion or γ subunit which are proteolytically removed within the secretory pathway (Prins et al. 2026).

Notably, while currently known natural yeast toxin-immunity pairs involve secreted toxins, both single-ORF and separate-ORF toxin-immunity pairs have been modified to only act *cis* toxic on the producer (Fig. 2B): For example, expression of the toxic α-subunit of the K2 toxin (which is part of a single-ORF toxin-immunity pair) leads to a *cis*-toxicity phenotype only, when expressed without the other subunits and with a nonnative secretion signal which does not contain the nested immunity factor (Prins and Billerbeck 2024). Moreover, the suicidal K2 α-subunit was functional in a broader range of culturing conditions (i.e., media pH and temperature) than the original, secreted K2 toxin (Prins and Billerbeck 2024). Further, the PaT, and DrT toxins can also be turned into systems that exhibit *cis* toxicity alone by expressing their toxic γ subunit without the N-terminal secretion signal (Butler et al. 1991; Gier et al. 2017; Klassen et al. 2014; Paluszynski et al. 2007).

To be able to deconvolute and test *cis* toxicity and *cis/trans* immunity, a combination of three assays can be used as a starting point, with detailed protocols published and (Prins and Billerbeck 2025a) (Fig. 2C). Those assays require inducible toxin expression and expression of the immunity factor from a separate plasmid. Further, to observe the phenotypes discussed below, it is required that a researcher has prior knowledge of the environment in which a toxin is active. For example, many single-ORF-encoded toxins are only active at low pH and ambient temperature. As such, the assay conditions need to be adjusted to these activity requirements. *Trans* toxicity can be assessed in a halo-assay where a droplet of a candidate yeast is placed on a lawn of target cells: A growth-inhibition halo indicates* trans* toxicity. The absence of a growth-inhibition halo indicates that the toxin is not—or not sufficiently—acting in the extracellular space to kill neighboring cells. *Trans* immunity can also be evaluated by a halo-assay, or by challenging otherwise sensitive cells that express a potential immunity factor directly with concentrated toxin collected from producer cultures. Normal growth indicates functional *trans* immunity. *Cis* toxicity can be observed as reduced fitness in a spot assay upon expression of an (engineered) toxin in the absence of co-expression of a cognate immunity factor. To ensure that growth inhibition is not simultaneously the result of a secreted active toxin, experimental conditions should result in a lack of *trans* toxicity, for example, by tuning media pH. *Cis* immunity can be tested by rescuing the suicidal phenotype described above by co-expression of an immunity factor. As mentioned earlier, *cis* and *trans* immunity might be established due to the same factor, or distinct ones. To infer the phenotype of a toxin from the previously described assays, their results should be combined (Fig. 2D). Despite the limitations of these assays and definitions (see Supplementary Note 1), we believe they are sufficient to distinguish toxicity and immunity phenotypes for practical applications and toxin comparisons.

### Single-ORF toxin-immunity pairs encoded on dsRNA satellites: coding mechanism, sequence-function relationships and mode of protection

Here we specifically discuss the toxins K1, K2, and K28 for which toxin and immunity factors are encoded within the same ORF, and for which it has been (partly) disentangled where in the ORF the immunity is encoded and how it protects the cell from intoxication.

**Fig. 3:**
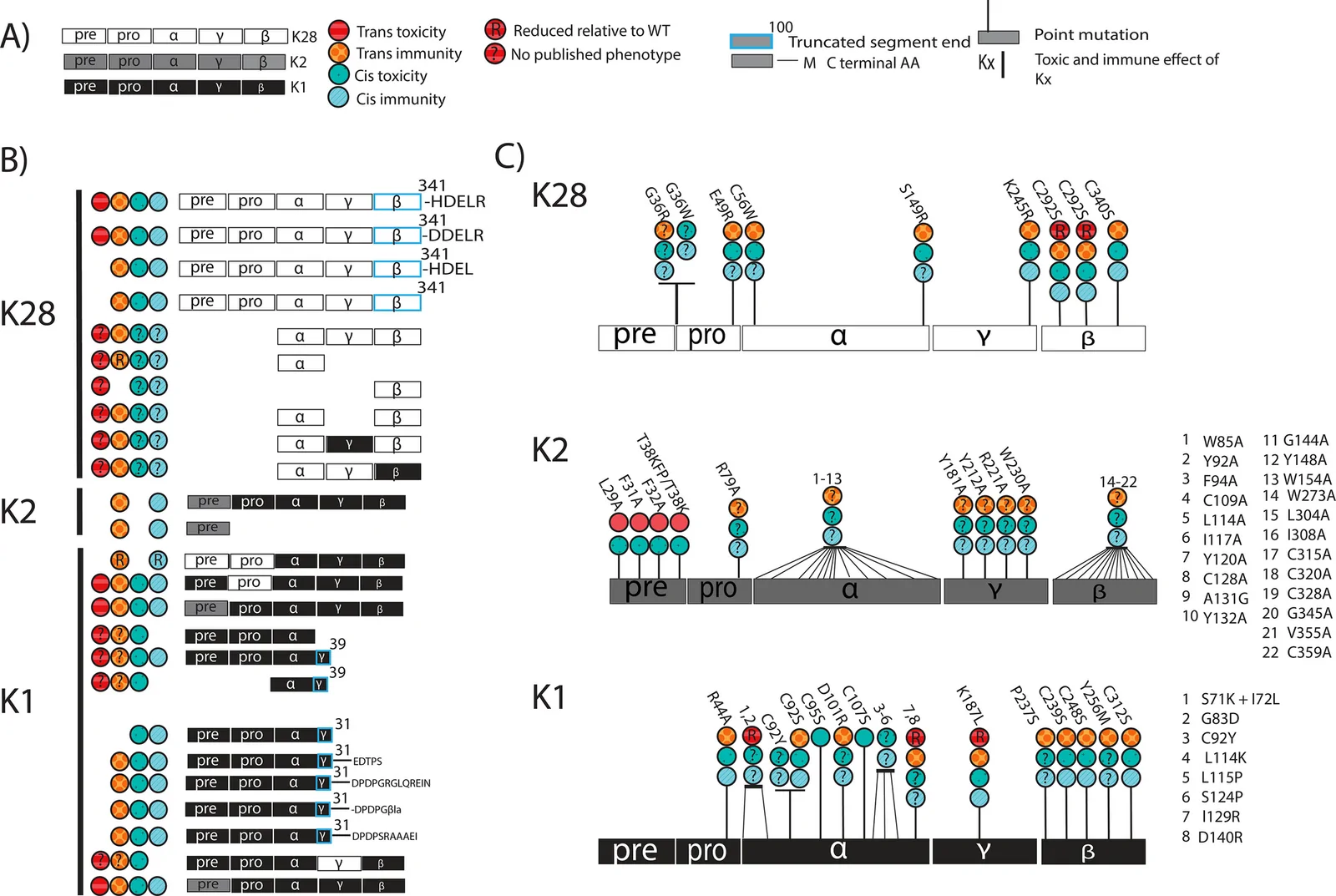
Expression of toxin derivatives has shaped our understanding of the sequence-function relationships of the killer toxins K1, K2, and K28. **A** The toxin sequences contain several distinct domains responsible for* cis *and *trans* immunity and toxicity. **B** Summary of relevant reported toxin chimeras and truncated variants which lost one of the various functions when compared with the wild-type toxin. Colored dots indicate the various functions (*cis/trans* toxicity; *cis/trans* immunity) and the absence of a colored dot indicates that the variant lost this phenotype, or that a phenotype is reduced or unknown. K28: *tTrans* toxicity is lost upon truncation of the toxin. *tTrans* immunity requires the presence of the α domain; however, sole expression of the α domain only provides reduced immunity (Breinig et al. 2006; Riffer et al. 2002; Suzuki et al. 2017). K2: The K2 preregion is required and sufficient for *trans* and *cis* immunity (Prins and Billerbeck 2024). K1: *tTrans* toxicity is lost in toxin truncations or upon substitution of the preregion with the K28 preregion. *trans* immunity is lost when the toxin γ domain is truncated to 31 amino acids but can be restored by extending the toxin with various amino acid sequences. *cCis* immunity is lost in the absence or substitution of the γ domain. The minimal sequence required for *cis* immunity consists of the preproregion, α domain and the first 31 amino acids of the γ domain. Removal of the preproregion leads to loss of *cis* immunity in toxin derivatives with smaller C-terminal truncations (Gier et al. 2020; Zhu et al. 1993). **C** Summary of relevant reported point mutations which resulted in the loss of one or several of the above-outlined functions of K28 (Riffer et al. 2002; Suzuki et al. 2017), K2 (Prins et al. 2024), and K1 (Gier et al. 2019).

All three toxins are encoded on dsRNA (M) satellites which are not capable of autonomous replication and instead rely on the presence of a dsRNA L-A-helper virus (Fig. 1A). The 4.6 kb L-A-helper virus encodes all components for transcription, packaging and replication of LA and M satellites (Aitmanaitė et al. 2021). Each M satellite contains a single ORF at the 5′ region encoding a preprotoxin precursor, followed by a poly-A stretch and a 3′ non-coding region. K1, K2 and K28 share no sequence homology but a similar precursor architecture (Fig. 3A), consisting of an N-terminal signal peptide (preregion), a proregion, and an α, γ and β subunit. The signal peptide guides the toxin precursor into the secretory pathway, where proteolytic cleavage leads to secretion of the mature α/β toxins, while the central γ subunit and the proregion are excised. For all toxins it was shown that the killing property is encoded by the α subunit, while the β subunit facilitates* trans* toxicity. Despite no sequence homology, K1 and K2 share structural similarities based on structure predictions and are suggested to belong to a common structural toxin family (Prins et al. 2024). Detailed information on the structure and function of these three *S. cerevisiae* killer toxins has been reviewed before (Creagh et al. 2026).

The single-ORF K28 toxin-immunity pair has functional analogies to the three later discussed VLE-encoded toxin-immunity pairs as it involves a secreted toxin that is taken up by the target (and producer) cells and acts intracellularly, causing irreversible G1/S cell cycle arrest. K28’s immunity is mediated by the unprocessed toxin precursor that neutralizes the retrotransported mature toxin intracellularly via binding, followed by binding-induced ubiquitination and proteasome degradation (Breinig et al. 2006).

More specifically, the K28 toxin and its immunity factor are encoded within a single 345-amino-acid preprotoxin-coding ORF on the M28 dsRNA satellite (Becker and Schmitt 2017). Mature, secreted K28 exhibits its *trans* toxicity by first binding to manoprotein in the target’s cell wall. The protein subsequently penetrates the cell wall and interacts with the receptor Erd2p via its HDEL motif (Becker and Schmitt 2017). This toxin-receptor complex is then endocytosed and retrotransported via the Golgi and the ER to the cytosol. In the cytosol, the K28 α subunit dissociates from the β subunit and diffuses into the nucleus where it causes irreversible G1/S cell cycle arrest. Retrograde toxin transport critically depends on the presence of a short amino-acid sequence (HDEL) at the C-terminus of the toxin. The same sequence is used by other toxins, such as cholera toxin, for retrotransport (Lencer 2003). Deletion or shortening of this sequence abolishes *trans* toxicity but retains the immunity encoded in the precursor (Eisfeld et al. 2000) (Fig. 3B).

Genetic studies involving the isolated expression of either one of the three subunits as well as synthetic chimeras revealed that the K28 α subunit is critical but not sufficient for immunity and that an extension at its C-terminus is needed Fig. 3B and Supplementary Fig. 2). Interestingly, it was shown that the sequence of this extension can be quite flexible: It can be the native γ subunit, but also synthetic chimeras with the α subunit fused to the β subunit, or to the γ subunit of the K1 toxin, provided immunity (Breinig et al. 2006). As such, a shorter version of the precursor can be expressed to yield an immunity module that does not show* trans* toxicity. Complete modularization of the original K28 toxin and immunity sequence into two independent ORFs does not seem possible with the current knowledge, as the immunity is encoded within the toxic domain, thus a *trans* toxicity module always encodes *cis* and* trans* immunity.

Mutational studies have revealed several critical residues within the toxin α and β subunit (Riffer et al. 2002; Suzuki et al. 2017) (Fig. 3C), focusing on Kex2p cleavage sites (GR_49_, SR_149_ and KR_245_ are essential) (Riffer et al. 2002) and cysteines (C_56_ within the α, and C_307_, C_333_ and C_340_ within β are essential for *trans* toxicity) (Suzuki et al. 2017). However, critical residues within the immunity region and its mutational tolerance have not been studied yet.

The single-ORF K2 toxin-immunity pair involves a secreted toxin that exhibits *trans* toxicity by acting on the membrane of target and producer cells. The K2 toxin and immunity factor are encoded as a 362 amino acid preprotoxin ORF on the M2 dsRNA satellite (Dignard et al. 1991). K2 first binds to the cell wall of target yeast via β−1,6-glucans, followed by postulated binding to its secondary plasma membrane receptor Kre1p (Lukša et al. 2015). Eventually, it is hypothesized to kill via an ionophoric disruption of membrane function due to observed morphological changes of cells after intoxication and its structural similarity to K1 (Martinac et al. 1990; Vadasz et al. 2000). A systematic deletion scan identified the immunity factor to be located within the secretion signal (preregion), which gets cleaved off while the toxin is secreted, protecting the cells from *cis *and *trans* intoxication by a yet unknown mechanism (Prins and Billerbeck 2024).

Specifically, deletion of the first 54 amino acids of K2 (regions 1–54) leads to loss of *cis* and *trans* immunity (Prins and Billerbeck 2024). Separate expression of this 54-residue immunity peptide provided protection against externally added K2 and against K2 variants that were secreted via an alternative secretion signal lacking the immunity function. Through separate expression of the immunity peptide and this K2 variant, a modular toxin-antitoxin system could be created. Further, substitution of the K1 signal peptide with the K2 immunity/signal peptide led to secretion of active K1 and immunity to both K1 and K2. In addition, an alanine scan indicated that most residues within the immunity peptide could be individually mutated without loss of *trans* immunity, but that a few positions may be important for immunity: L29A, F31A, and F32A (Prins et al. 2024) (Fig. 3C). The same scan revealed critical residues for *trans* and *cis* toxicity, such as several cysteine residues, Kex2p cleavage sites and several hydrophobic residues. A few alanine substitutions also led to K2 variants with higher potency and changed targeting specificity.

The single-ORF K1 toxin-immunity pair—similar to K2—also involves a secreted toxin that exhibits *trans* toxicity by acting on the membrane of target and producer cells. The K1 toxin and immunity factor are encoded on a 316 preprotoxin ORF which was detected on the M1 dsRNA satellite (Russell et al. 1997). Like K2, K1 first binds to the cell wall of target yeast via β−1,6-glucans, followed by binding to its secondary plasma membrane receptor Kre1p (Novotna et al. 2004). Despite similarities in cell wall and plasma membrane interactions between K1 and K2, a deletion study also pointed to various different gene products being critical for target-cell interaction (Novotna et al. 2004). The *cis* and *trans* toxic effect of K1 is due to pore formation (Bussey 1991). The exact molecular mechanism of K1 immunity is not known; however, it is speculated to require processing in the secretory pathway (Vališ et al. 2006). Studies on K1 derivatives have shown that immunity requires an ORF containing the preproregion, the α-domain and an extension into the γ-domain. Substitution of the whole preproregion leads to loss of *trans* immunity although the signal peptide can be replaced by the K2 or K28 signal peptide (Fig. 3B) (Gier et al. 2020; Prins and Billerbeck 2025b). Mutations in the two α-domain cysteines C95 and C108 cause loss of immunity (Gier et al. 2019). No noticeable loss of K1* cis* toxicity was observed when mutating these cysteine residues (Fig. 3C). A truncated K1 derivative containing 31 N-terminal amino acids of the γ-domain was sufficient for *cis* immunity whereas* trans* immunity required the presence of eight additional residues (Zhu et al. 1993). Surprisingly, substitution of the K1 γ-domain with the K28 γ-domain still led to immunity despite the low sequence similarity (Gier et al. 2020).

### Separate ORF toxin-immunity pairs encoded on VLEs: coding mechanism and mode of protection

Here we exemplify the three most studied VLE-encoded toxins: zymocin from *K. lactis* (Tokunaga et al. 1987a), PaT from *P. acacia* (Paluszynski et al. 2007), and DrT from *D. robertsiae* (Klassen and Meinhardt 2002)*.* Toxin production and toxin-immunity in these yeasts are associated with the presence of a pair of linear cytoplasmic dsDNA elements that show similar gene organization (Fig. 1B). One element encodes the toxin subunits (two ORFs) and immunity (one ORF) and the other provides essential functions for cytoplasmic maintenance such as replication, transcription, and transcript processing. The dsDNA elements are called pGKL1 and pGKL2 for zymocin production (Tokunaga et al. 1987a), Pac1-2 and Pac1-1 for PaT production (Paluszynski et al. 2007), and pWR1A and pWR1B for DrT production (Klassen and Meinhardt 2002). These cytoplasmic elements were eventually termed virus-like elements (VLE) as their polymerases show phylogenetic proximity to viral ones (Satwika et al. 2012) and because their proposed mode of replication, which involves protein priming, is typically found in viruses (Jeske et al. 2007).

The three toxins are functional homologs and kill target cells by translocation into the susceptible cell’s cytoplasm and inducing cell death by cleaving specific tRNA substrates. PaT and DrT target tRNA^Gln^_UUG_ while zymocin targets tRNA^Glu^_UUC_ (Klassen et al. 2008, 2014; Lu et al. 2005). The toxins are heterotrimeric with an α, β and γ subunit, with α and β being encoded in a single ORF and γ being encoded in a separate ORF (Strak et al. 1990) (Fig. 1B). The γ subunit (also called γ-toxin) is the tRNA-cleaving ribonuclease which acts intracellularly after uptake. The co-encoded α and β subunits are proteolytically processed in the secretory pathway into two separate polypeptide chains and have chitin-binding and chitinase activity, typically containing a chitin-binding domain (ChtBD1), a glycoside hydrolase GH18 domain (Glyco_18), and a LysM *N*-acetylglucosamine-binding domain (LysM) (Heneghan et al. 2024a, b). These α/β units bind chitin in the target’s cell wall and deliver the γ-toxin close to the cell surface for uptake, thus the α/β subunits are essential for *trans*-toxicity. Both γ-toxin and the α/β chitinase carry N-terminal secretion signals and are exported by the killer cell. As mentioned earlier, expressing the γ-toxin alone without an N-terminal secretion signal can turn it into a *cis*-toxic subunit only acting on the producer.

Self-intoxication is avoided because each VLE also encodes a non-secreted immunity protein that neutralizes toxin molecules re-entering the producer cell (Klassen et al. 2014; Paluszynski et al. 2007; Strak et al. 1990). The PaT immunity factor is encoded on PaORF4 (363 amino acids) (Paluszynski et al. 2007), the zymocin immunity factor is encoded on KlORF3 (452 amino acids) (Strak et al. 1990), and the DrT *cis*-immunity factor is encoded on DrORF5 (353 amino acids) (Fig. 1B).

Structural analysis has shown that PaT immunity is mediated by direct intracellular binding of the cognate immunity factor (PaORF4) to the toxin (Chakravarty et al. 2014). Although structural confirmation is lacking for other pairs, it was shown that DrORF5 and KlORF3 also neutralize their toxin inside the cell rather than by blocking docking or entry (Klassen et al. 2014). Given the approximately 50% sequence identity between PaORF4 and DrORF5 and that these toxins are functional homologs, it is likely that they also neutralize via binding. Accordingly, all three immunity factors likely confer both *cis* and *trans* protection.

### Cross immunity versus orthogonal toxin-immunity pairs

One open question about the co-encoded toxin-immunity pairs is their level of orthogonality toward each other, meaning the question whether one factor can provide immunity against several distinct toxins or whether the interaction is specific. We define cross-protection as the ability of a single immunity factor to protect an otherwise sensitive host against a cognate and noncognate toxin. Only a few cases have been systematically studied: For the VLE-encoded separate-ORF toxin-immunity pairs DrT/DrORF5 and PaT/PaORF4 different levels of orthogonality were shown in expression studies, where immunity and toxin ORFs were co-expressed in a heterologous host (*S. cerevisiae*) and *cis* and *trans* toxicity were assessed. While DrORF5 protected only against its cognate toxin DrT, PaORF4 provided cross-immunity against both toxins, however less effective against DrT. Sequence similarity between the two immunity factors is 50%, suggesting that only key residues are required for immunity and determine specificity (Klassen et al. 2014).

Given this type of co-expression assay is relatively straightforward, it could be feasible to also test orthogonality of other zymocin-like toxins and immunity factors recently discovered in the before-discussed bioinformatics survey (Heneghan et al. 2024a, b).

With respect to orthogonality in single-ORF toxin-immunity pairs, it was shown that co-encoded immunity factors of K1 and K2 do not provide significant cross-protection in a similar expression experiment in a heterologous susceptible host (Prins and Billerbeck 2024).

Also, other surveys with wild toxin-producing isolates indicate a lack of cross-immunity: Wild K1 toxin producers were not resistant to a K1-like killer toxin producer (33% amino acid similarity). Other K1-like toxins producers were not cross-resistance despite their toxins sharing 22–45% sequence similarity (Fredericks et al. 2021). However, we lack knowledge on the specific immunity factors of these K1-like toxins and whether they are co-encoded or due to genomic resistance.

Further, various natural killer yeast isolates producing the toxins K1, K2, K28, K45, K62, and K74 were challenged against each other, resulting in differences in halo size across strains harboring the different killer toxins (Fredericks et al. 2021; Rodriguez-Cousino et al. 2022; Rodríguez-Cousiño et al. 2017). This indicates potential cross-resistance; however, it could also be caused by differences in sensitivity, as killing activity against cured strains was not tested (Fredericks et al. 2021; Rodriguez-Cousino et al. 2022; Rodríguez-Cousiño et al. 2017).

### Applications for toxin-immunity pairs

Bacterial toxin-antitoxin systems have been explored in synthetic biology as mechanisms for plasmid addiction for stable fermentations, as inducible biocontainment modules, and as selectable markers in cloning vectors (Lin et al. 2023; Qiu et al. 2022). Yeast toxin-immunity pairs could find analogous applications.

In terms of plasmid addiction, classically, bacterial plasmid-addiction systems exploit differential stability with a long-lived toxin and an unstable antitoxin (Lin et al. 2023; Qiu et al. 2022): When a plasmid is lost, antitoxin synthesis ceases, and the antitoxin pool rapidly decays. The persisting toxin subsequently kills the cell, thus selecting against plasmid-free progenies (Lin et al. 2023). A similar selection mechanism has been proposed for the maintenance of extrachromosomal elements of yeast, such as the dsRNA satellites and VLEs (Boynton 2019). Here, loss of a dsRNA satellite can be lethal not because of differential stability, but because neighboring cells continue to secrete toxin; a cell that loses the element simultaneously loses both toxicity and immunity and is consequently susceptible to its neighbors (Boynton 2019; Rowley 2017).

For several applications of toxin-immunity pairs, it can be useful to clone and express the toxin and the immunity separately. For single-ORF toxin-immunity pairs, a split version has only been engineered for K2. A central question for achieving splitting is which region of the ORF encodes the toxin versus the immunity function and whether it is encoded in a modular way that would allow splitting the ORF. Early immunity mapping efforts relied on site-directed mutagenesis and creating domain chimeras for K1, K2 and K28 (Fig. 3B). For K1, this revealed that only defined stretches within the α and γ domains were required for activity, while for K28 an extended α subunit was necessary and that toxin and immunity are nested (Zhu et al. 1993). Advances in DNA synthesis, high-throughput assaying, and next-generation sequencing have made it more affordable to create and evaluate systematic mutational scanning and deletion scanning libraries (Maciá Valero et al. 2025; Prins and Billerbeck 2025a). Recently performed for K2, this revealed a short immunity peptide encoded within the secretion signal (Prins and Billerbeck 2024), further modularization eventually yielded a fully split toxin-immunity pair with separate ORFs coding for the toxin and immunity functions (Prins and Billerbeck 2024).

Separate expression of toxin and immunity factors obviously already occurs in the known VLEs. The high A/T content of VLE immunity factors has been proposed to act as a nuclear expression barrier (Kast et al. 2015), preventing capture of the immunity gene by the nuclear genome. Otherwise, a chromosomal copy would allow cells to survive a loss of the cytoplasmic VLE, as the immunity factor would be retained despite loss of the VLE-encoded gene (Kast et al. 2015; Wickner and Edskes 2015). As such, the natural toxin-immunity pairs discussed herein could have potential to be repurposed and directly tested as plasmid-addiction systems. Notably, engineered VLEs independent on the toxins have been used in synthetic biology for orthogonal directed evolution (OrthoRep mutagenesis system (Ravikumar et al. 2018)).

In addition, engineered intracellular toxin-immunity pairs could be adapted to bacterial-like plasmid-addiction systems by rendering the immunity factor unstable (e.g., via degradation tags (Nishimura and Kanemaki 2014)) and tuning the degradation dynamics to allow for addiction. Further insight into the stoichiometric dynamics of toxin-immunity pairs will be required to create stable addiction systems. It has been shown that K1 toxicity can be lost in a population of immune cells subsequently leading to loss of immunity as well (Buskirk et al. 2020). These intracellular pairs could also be harnessed for biocontainment by placing immunity or toxin expression under a conditional promoter present only in the fermentation environment; upon escape, the input is lost, antitoxin production stops, and the toxin eliminates escapees. These yeast toxin-based containment systems could be used to build orthogonal multiplexed control (as proposed (Pavão et al. 2023)), in tandem with existing metabolic biocontainment strategies such as auxotrophies (Pavão et al. 2023) or engineered small-molecules-dependent safeguards (Agmon et al. 2017). Notably, bacterial toxin-antitoxin systems have also been explored in yeast for biocontainment (Pavão et al. 2023).

Of course, prior to utilizing newly engineered toxin-antitoxin systems for plasmid-addiction or biocontainment, rigorous analysis for the acquisition of genomic resistance via mutations would need to be performed to assess the frequency of occurrence of escape mutants.

Next to plasmid-addiction and biocontainment, the expanding toolkit of secreted and intracellularly acting toxins variants and their cognate immunity factors offer a means to build synthetic yeast communities. Synthetic microbial ecology seeks to design controlled ecosystems for both mechanistic insight and technological applications, such as more robust consortia for industrial bioprocesses (Jiang et al. 2025; Matuszyńska et al. 2024). Studies employing engineered killer-toxin-secreting yeast have already begun to reveal how social interactions shape the evolutionary dynamics of microbial populations (Giometto et al. 2021), and their complex ecology yet tractable genetics suggests them as good model systems for ecological questions (Travers-Cook et al. 2023), or for programmable bottom-up microbial community design (Matuszyńska et al. 2024).

## Conclusion

Extrachromosomally co-encoded toxin-immunity pairs in yeast are found on two classes of cytoplasmic elements: dsRNA satellites and dsDNA VLEs (Schaffrath et al. 2018). Two architectures occur: First, single-ORF systems on dsRNA satellites, where a preproprotein harbors both toxin and immunity regions (typified by K1, K2, and K28 among others) (Dignard et al. 1991; Russell et al. 1997; Schmitt 1995). Second, separate-ORF systems on VLEs, where distinct genes encode the secreted toxin subunit and a dedicated, non-secreted immunity protein (typified PaT/PaORF4, zymocin/KlORF3, DrT/DrORF5) (Klassen and Meinhardt 2002; Paluszynski et al. 2007; Strak et al. 1990). Interestingly, all known pairs are encoded on split extrachromosomal element systems, with the toxin-immunity pair being co-encoded on one element which cannot replicate (except for zymocin which is encoded on a VLE which replicates itself) (Klassen and Meinhardt 2002) and relying on a separate autonomous element for ORF expression and replication (Klassen and Meinhardt 2002). This has sparked the interpretation that these toxin-immunity pairs serve the purpose of self-selection for the genetic element they are encoded on (Boynton 2019; Rowley 2017; Wickner and Edskes 2015), especially given the reported nuclear expression barrier for some immunity proteins prevents the pair leaving its genetic context (Kast et al. 2015). This natural purpose substantiates their potential usefulness as synthetic toxin–antitoxin systems for biotechnology. However, it is not yet completely understood how genomic and extrachromosomal immunity elements are maintained in natural populations and over what timescales immunity factors and toxins mutate relative to each other.

Mechanistically, the protection mechanism is established only for the intracellularly acting pairs where the immunity factors neutralize their cognate toxins by directly binding in the cytosol, analogous to bacterial toxin-antitoxin systems historically used in applications (Jurėnas et al. 2022; Becker and Schmitt 2017). For the membrane-acting toxins K1 and K2, their mechanism of protection remains unknown.

### Discovery, cloning, and lessons from nuclear expression

Curing assays (Fredericks et al. 2021; Vijayraghavan et al. 2023) and dsRNA (Crabtree et al. 2019) and VLE isolation (Połomska et al. 2021), followed by sequencing, have established the extrachromosomal encoding of these pairs. Heterologous expression from nuclear plasmids is straightforward for dsRNA single-ORF systems (Dignard et al. 1991; Russell et al. 1997; Schmitt 1995) and achievable for VLE systems once A/T-content has been recoded to overcome nuclear expression barriers (Kast et al. 2015). VLEs can also be engineered in the cytosol via in vivo homologous recombination to generate engineered hybrid VLEs (Schickel 1996).

In addition, heterologous expression of various toxins was possible, indicating that these systems could be applied in yeasts beyond their natural origin: (Giesselmann et al. 2017; Kast et al. 2014; Wemhoff et al. 2014).

### *Cis/trans* toxicity and immunity

To use the yeast toxin-immunity pairs for engineering, the* cis/trans* framework helps to disentangle toxicity and immunity during toxin production and against extracellular toxin activity. The central question is if *cis* immunity to the intracellular (precursor) toxin is the same as *trans* immunity to the external mature toxin. Engineered *cis*-only toxin variants (Fig. 2B) in combination with three assays (Fig. 2C) have helped to disentangle different scenarios and could also be helpful to systematically design, build and test new *cis*- and *trans-*acting systems.

VLE-encoded immunity factors function intracellularly and, as such, inherently provide *cis* and *trans* protection as they neutralize the intracellular toxin during production and after retrotransport.

For toxins like K1 and K2, where the mechanism of immunity is not yet understood on a molecular level, also the question on *cis *and *trans* protection mechanisms remains open.

### Next steps for application: modularization and generation of suicidal and fratricidal toxin-immunity pairs

A central opportunity emerges from the evidence for modularization of the discussed yeast toxin-immunity systems: namely, creating separate parts encoding intracellular toxin production (*cis* toxicity), toxin secretion (*trans* toxicity), and neutralization of both (*cis* and* trans* immunity) and the fact that these parts can be combined to yield either intracellularly acting bacteria-like toxin-antitoxin modules or extracellularly acting community-interaction systems.

It has been shown that intracellular *cis-*acting modules can be constructed by removing *trans*-toxic domains thus confining toxicity to the producer cell (Fig. 2B), (Prins and Billerbeck 2024; Klassen et al. 2014).

Extracellular-acting systems are readily built by using the native secreted toxins and their immunity. Because *cross*-immunity among unrelated pairs appears limited, different toxins could be combined as orthogonal channels to build user-defined community structures or multiplexed biocontainment. However, rigorous testing for orthogonality and further understanding of the features that dictate specificity and orthogonality are required.

Several design principles and experiment types have shown to be useful to generate either system: First, minimal immunity units can be determined via deletion and alanine scans (Prins et al. 2026; Prins and Billerbeck 2024). Second, for VLE-encoded toxins, adding or not adding a secretion signal enables a toxin variant to act in *cis* or *trans*. For dsRNA-encoded toxins, removing the domains facilitating *trans* toxicity enables a toxin variant to act in *cis*. Third, plasmid expression barriers for VLE immunity genes (often driven by extreme A/T content) can be overcome through codon optimization to enable intracellular neutralization in nuclear expression contexts (Kast et al. 2015). Fourth, *cis* and *trans* phenotypes can be disentangled with a triad of assays that are in theory high-throughput and can be used during engineering and DBTL cycling (Design Build Test Learn) (Fig. 2C) (Prins and Billerbeck 2025a): Halo-assays for *trans* toxicity, exogenous toxin challenges for *trans* immunity in immunity-expressing strains, and suicidal-phenotype rescue for co-expressed *cis* toxicity/*cis* immunity testing when *trans* toxicity is disabled.

By deliberately choosing between* cis*-toxic and *trans*-toxic configurations, engineers could potentially tailor yeast toxin-immunity systems to context: *cis*-toxin/immunity systems could be mainly used to regulate the producer’s fate, for example as plasmid-addiction systems or for biocontainment. *Trans*-toxic systems can help shape, stabilize, or control the surrounding microbial community—examples including: synthetic yeast communities and the production of toxins within systems prone to fungal contamination.

For building these systems, a deeper understanding of the forces that counteract *cis* and* trans* toxicity helps to assess system robustness: Those could include genetic forces such as mutations leading to resistance, biological forces such as dedicated immunity factors and proteases that neutralize or degrade the toxin and physicochemical forces such as temperature and pH that inactivate the toxin.

## Supplementary Information

Below is the link to the electronic supplementary material.

ESM 1 (DOCX 385 KB)

## Data Availability

Not applicable; this manuscript does not contain new data.

## References

[CR1] Agmon N, Tang Z, Yang K, Sutter B, Ikushima S, Cai Y, Caravelli K, Martin JA, Sun X, Choi WJ, Zhang A, Stracquadanio G, Hao H, Tu BP, Fenyo D, Bader JS, Boeke JD (2017) Low escape-rate genome safeguards with minimal molecular perturbation of *Saccharomyces cerevisiae*. Proc Natl Acad Sci U S A 114(8):1621250114. 10.1073/pnas.1621250114 10.1073/pnas.1621250114 PMC5338387 28174266

[CR2] Aitmanaitė L, Konovalovas A, Medvedevas P, Servienė E, Serva S (2021) Specificity determination in *Saccharomyces cerevisiae* killer virus systems. Microorganisms 9(2):236. 10.3390/microorganisms9020236 33498746 10.3390/microorganisms9020236 PMC7912047

[CR3] Andreev I, Laidlaw KME, Giovanetti SM, Urtecho G, Shriner D, Bloom JS, MacDonald C, Sadhu MJ (2023) Discovery of a rapidly evolving yeast defense factor, KTD1, against the secreted killer toxin K28. Proc Natl Acad Sci U S A 120(8):Article 8. 10.1073/pnas.2217194120 10.1073/pnas.2217194120 PMC9974470 36800387

[CR4] Arbulu S, Kjos M (2024) Revisiting the multifaceted roles of bacteriocins: the multifaceted roles of bacteriocins. Microb Ecol 87(1):41. 10.1007/s00248-024-02357-4 38351266 10.1007/s00248-024-02357-4 PMC10864542

[CR5] Becker B, Schmitt M (2017) Yeast killer toxin K28: biology and unique strategy of host cell intoxication and killing. Toxins Basel 9(10):333. 10.3390/toxins9100333 29053588 10.3390/toxins9100333 PMC5666379

[CR6] Billerbeck S, Walker RSK, Pretorius IS (2024) Killer yeasts: expanding frontiers in the age of synthetic biology. Trends Biotechnol 0(0):Article 0. 10.1016/j.tibtech.2024.03.003 10.1016/j.tibtech.2024.03.003 38575438

[CR7] Boynton PJ (2019) The ecology of killer yeasts: interference competition in natural habitats. Yeast 36(8):473–485. 10.1002/yea.3398 31050852 10.1002/yea.3398

[CR8] Breinig F, Sendzik T, Eisfeld K, Schmitt MJ (2006) Dissecting toxin immunity in virus-infected killer yeast uncovers an intrinsic strategy of self-protection. Proc Natl Acad Sci U S A 103(10):Article 10. 10.1073/pnas.0510070103 10.1073/pnas.0510070103 PMC1533781 16505373

[CR9] Buskirk SW, Rokes AB, Lang GI (2020) Adaptive evolution of nontransitive fitness in yeast. Elife 9:e62238. 10.7554/eLife.62238 33372653 10.7554/eLife.62238 PMC7886323

[CR10] Bussey H (1991) K1 killer toxin, a pore-forming protein from yeast. Mol Microbiol 5(10):2339–2343. 10.1111/j.1365-2958.1991.tb02079.x 1724277 10.1111/j.1365-2958.1991.tb02079.x

[CR11] Butler AR, Porter M, Stark MJR (1991) Intracellular expression of *Kluyveromyces lactis* toxin γ subunit mimics treatment with exogenous toxin and distinguishes two classes of toxin‐resistant mutant. Yeast 7(6):617–625. 10.1002/yea.320070610 1767590 10.1002/yea.320070610

[CR12] Chakravarty AK, Smith P, Jalan R, Shuman S (2014) Structure, mechanism, and specificity of a eukaryal tRNA restriction enzyme involved in self-nonself discrimination. Cell Rep 7(2):339–347. 10.1016/j.celrep.2014.03.034 24726365 10.1016/j.celrep.2014.03.034 PMC4121121

[CR13] Crabtree AM, Kizer EA, Hunter SS, Van Leuven JT, New DD, Fagnan MW, Rowley PA (2019) A rapid method for sequencing double-stranded RNAs purified from yeasts and the identification of a potent K1 killer toxin isolated from *Saccharomyces cerevisiae*. Viruses 11(1):70. 10.3390/v11010070 30654470 10.3390/v11010070 PMC6356530

[CR14] Crabtree AM, Taggart NT, Lee MD, Boyer JM, Rowley PA (2023) The prevalence of killer yeasts and double-stranded RNAs in the budding yeast *Saccharomyces cerevisiae*. FEMS Yeast Res 23:foad046. 10.1093/femsyr/foad046 37935474 10.1093/femsyr/foad046 PMC10664976

[CR15] Creagh JW, Reetz DC, Givens LL, Coss SA Jr, RB, Patel JS, Rodrigues A, Ytreberg FM, Rowley PA (2026) A comprehensive structural and functional analysis of Saccharomyces killer toxins 10.3390/toxins18050235 PMC13211469 42188637

[CR16] Creagh JW, Rolfsmeier M, Evans KJ, Bizarria R, Reetz DC, Badigian TJ, Fredericks LR, Hasenoehrl AM, Brown AP, Graves BM, Alexander CK, Rodrigues A, Stoffregen EP, Patel JS, Ytreberg FM, Rowley PA (2025) The *Saccharomyces* killer toxin K62 is a protein of the aerolysin family. mBio 16(12):e01425-25. 10.1128/mbio.01425-25 41217184 10.1128/mbio.01425-25 PMC12691639

[CR17] Crucitti D, Chiapello M, Oliva D, Forgia M, Turina M, Carimi F, La Bella F, Pacifico D (2021) Identification and molecular characterization of novel mycoviruses in *Saccharomyces* and non-*Saccharomyces* yeasts of oenological interest. Viruses 14(1):52. 10.3390/v14010052 35062256 10.3390/v14010052 PMC8778689

[CR18] Dignard D, Whiteway M, Germain D, Tessier D, Thomas DY (1991) Expression in yeast of a cDNA copy of the K2 killer toxin gene. Mol Gen Genet 227(1):127–136. 10.1007/BF00260717 2046653 10.1007/BF00260717

[CR19] Duperray M, François J-M, Capp J-P (2023) Tuning the expression of the bacterial relBE toxin–antitoxin system in *Saccharomyces cerevisiae* allows characterizing the subsequent growth inhibition. FEMS Yeast Res 23:foad009. 10.1093/femsyr/foad009 36722160 10.1093/femsyr/foad009

[CR20] Eisfeld K, Riffer F, Mentges J, Schmitt MJ (2000) Endocytotic uptake and retrograde transport of a virally encoded killer toxin in yeast. Mol Microbiol 37(4):926–940. 10.1046/j.1365-2958.2000.02063.x 10972812 10.1046/j.1365-2958.2000.02063.x

[CR21] Finkler A, Peery T, Tao J, Bruenn J, Koltin I (1992) Immunity and resistance to the KP6 toxin of *Ustilago maydis*. Mol Gen Genet 233(3):395–403. 10.1007/BF00265436 1620096 10.1007/BF00265436

[CR22] Fredericks LR, Lee MD, Crabtree AM, Boyer JM, Kizer EA, Taggart NT, Roslund CR, Hunter SS, Kennedy CB, Willmore CG, Tebbe NM, Harris JS, Brocke SN, Rowley PA (2021) The species-specific acquisition and diversification of a K1-like family of killer toxins in budding yeasts of the Saccharomycotina. PLoS Genet 17(2):Article 2. 10.1371/JOURNAL.PGEN.1009341 10.1371/journal.pgen.1009341 PMC7888664 33539346

[CR23] Gier S, Schmitt MJ, Breinig F (2017) Expression of K1 toxin derivatives in *Saccharomyces cerevisiae* mimics treatment with exogenous toxin and provides a useful tool for elucidating K1 mechanisms of action and immunity. Toxins Basel 9(11):Article 11. 10.3390/toxins9110345 10.3390/toxins9110345 PMC5705960 29076990

[CR24] Gier S, Lermen M, Schmitt MJ, Breinig F (2019) Substitution of cysteines in the yeast viral killer toxin K1 precursor reveals novel insights in heterodimer formation and immunity. Sci Rep 9(1):13127. 10.1038/s41598-019-49621-z 31511600 10.1038/s41598-019-49621-z PMC6739482

[CR25] Gier S, Schmitt MJ, Breinig F (2020) Analysis of yeast killer toxin K1 precursor processing via site-directed mutagenesis: implications for toxicity and immunity. mSphere 5(1):Article 1. 10.1128/msphere.00979-19 10.1128/mSphere.00979-19 PMC7021474 32051241

[CR26] Giesselmann E, Becker B, Schmitt MJ (2017) Production of fluorescent and cytotoxic K28 killer toxin variants through high cell density fermentation of recombinant *Pichia pastoris*. Microb Cell Fact 16(1):228. 10.1186/s12934-017-0844-0 29258515 10.1186/s12934-017-0844-0 PMC5735513

[CR27] Giometto A, Nelson DR, Murray AW (2021) Antagonism between killer yeast strains as an experimental model for biological nucleation dynamics. eLife 10:e62932. 10.7554/eLife.62932 34866571 10.7554/eLife.62932 PMC8730724

[CR28] Heintel T, Zagorc T, Schmitt MJ (2001) Expression, processing and high level secretion of a virus toxin in fission yeast. Appl Microbiol Biotechnol 56(1–2):165–172. 10.1007/s002530100633 11499925 10.1007/s002530100633

[CR29] Heneghan PG, Salzberg LI, Cinnéide EÓ, Dewald JA, Weinberg CE, Wolfe KH (2024a) Ancient origin and high diversity of zymocin-like killer toxins in the budding yeast subphylum. Evol Biol. 10.1101/2024.09.27.615389 10.1073/pnas.2419860122 PMC11848437 39928860

[CR30] Heneghan PG, Salzberg LI, Wolfe KH (2024b) Zymocin-like killer toxin gene clusters in the nuclear genomes of filamentous fungi. Evol Biol. 10.1101/2024.10.25.620242 10.1016/j.fgb.2024.103957 39756571

[CR31] Jeske S, Meinhardt F, Klassen R (2007) Extranuclear inheritance: virus-like DNA-elements in yeast. In: Esser K, Löttge U, Beyschlag W, Murata J (eds) Progress in botany. Springer, Berlin Heidelberg, pp 98–129. 10.1007/978-3-540-36832-8_5

[CR32] Jiang W, Wang S, Gu F, Yang X, Qi Q, Liang Q (2025) Advances in synthetic microbial ecosystems approach for studying ecological interactions and their influencing factors. Eng Microbiol 5(2):100205. 10.1016/j.engmic.2025.100205 41982405 10.1016/j.engmic.2025.100205 PMC12967826

[CR33] Jurėnas D, Fraikin N, Goormaghtigh F, Van Melderen L (2022) Biology and evolution of bacterial toxin–antitoxin systems. Nat Rev Microbiol 20(6):335–350. 10.1038/s41579-021-00661-1 34975154 10.1038/s41579-021-00661-1

[CR34] Kamper J (1989) In vivo construction of linear vectors based on killer plasmids from *Kluyveromyces lactis*: selection of a nuclear gene results in attachment of telomeres. Mol Cell Biol. 10.1128/mcb.9.9.3931 2779572 10.1128/mcb.9.9.3931-3937.1989 PMC362455

[CR35] Kast A, Klassen R, Meinhardt F (2014) rRNA fragmentation induced by a yeast killer toxin. Mol Microbiol 91(3):606–617. 10.1111/mmi.12481 24308908 10.1111/mmi.12481

[CR36] Kast A, Voges R, Schroth M, Schaffrath R, Klassen R, Meinhardt F (2015) Autoselection of cytoplasmic yeast virus like elements encoding toxin/antitoxin systems involves a nuclear barrier for immunity gene expression. PLoS Genet 11(5):Article 5. 10.1371/JOURNAL.PGEN.1005005 10.1371/journal.pgen.1005005 PMC4431711 25973601

[CR37] Klassen R, Meinhardt F (2002) Linear plasmids pWR1A and pWR1B of the yeast *Wingea robertsiae* are associated with a killer phenotype. Plasmid 48(2):142–148. 10.1016/S0147-619X(02)00101-4 12383731 10.1016/s0147-619x(02)00101-4

[CR38] Klassen R, Meinhardt F (2007) Linear protein-primed replicating plasmids in eukaryotic microbes. In: Meinhardt F, Klassen R (eds) Microbial linear plasmids, vol vol. 7. Springer, Berlin Heidelberg, pp 187–226. 10.1007/7171_2007_095

[CR39] Klassen R, Paluszynski JP, Wemhoff S, Pfeiffer A, Fricke J, Meinhardt F (2008) The primary target of the killer toxin from *Pichia acaciae* is tRNAGln. Mol Microbiol 69(3):681–697. 10.1111/j.1365-2958.2008.06319.x 18532979 10.1111/j.1365-2958.2008.06319.x

[CR40] Klassen R, Kast A, Wünsche G, Paluszynski JP, Wemhoff S, Meinhardt F (2014) Immunity factors for two related tRNAGln targeting killer toxins distinguish cognate and non-cognate toxic subunits. Curr Genet 60(3):Article 3. 10.1007/s00294-014-0426-1 10.1007/s00294-014-0426-1 24719080

[CR41] Kristoffersen P, Jensen GB, Gerdes K, Piškur J (2000) Bacterial toxin-antitoxin gene system as containment control in yeast cells. Appl Environ Microbiol 66(12):5524–5526. 10.1128/AEM.66.12.5524-5526.2000 11097943 10.1128/aem.66.12.5524-5526.2000 PMC92497

[CR42] Kurzweilová H, Sigler K (1993) Factors affecting the susceptibility of sensitive yeast cells to killer toxin K1. Folia Microbiol 38(6):524–526. 10.1007/BF02814408 8150399 10.1007/BF02814408

[CR43] Larsen M, Meinhardt F (2000). Kluyveromyces lactis killer system: identification of a new gene encoded by pGKL2. Curr Genet 38(5):271–5. 10.1007/s002940000167 10.1007/s002940000167 11191211

[CR44] Lencer W (2003) The intracellular voyage of cholera toxin: going retro. Trends Biochem Sci 28(12):639–645. 10.1016/j.tibs.2003.10.002 14659695 10.1016/j.tibs.2003.10.002

[CR45] Lin J, Guo Y, Yao J, Tang K, Wang X (2023) Applications of toxin–antitoxin systems in synthetic biology. Eng Microbiol 3(2):100069. 10.1016/j.engmic.2023.100069 39629251 10.1016/j.engmic.2023.100069 PMC11610964

[CR46] Lowes KF, Shearman CA, Payne J, MacKenzie D, Archer DB, Merry RJ, Gasson MJ (2000) Prevention of yeast spoilage in feed and food by the yeast mycocin HMK. Appl Environ Microbiol 66(3):1066–1076. 10.1128/AEM.66.3.1066-1076.2000 10698773 10.1128/aem.66.3.1066-1076.2000 PMC91944

[CR47] Lu J, Huang B, Esberg A, Johansson MJ, Byström AS (2005) The Kluyveromyces lactis gamma-toxin targets tRNA anticodons. RNA (New York, N.Y.) 11(11):1648–1654. 10.1261/rna.2172105 10.1261/rna.2172105 PMC1370851 16244131

[CR48] Lukša J, Podoliankaitė M, Vepštaitė I, Strazdaitė-Žielienė Ž, Urbonavičius J, Servienė E (2015) Yeast β-1,6-glucan is a primary target for the *Saccharomyces cerevisiae* K2 toxin. Eukaryot Cell 14(4):406–414. 10.1128/EC.00287-14 25710965 10.1128/EC.00287-14 PMC4385797

[CR49] Maciá Valero A, Prins RC, de Vroet T, Billerbeck S (2025) Combining oligo pools and Golden Gate cloning to create protein variant libraries or guide RNA libraries for CRISPR applications. In: Schindler D (ed) *Golden Gate cloning: methods and protocols*. Springer US, pp 265–295. 10.1007/978-1-0716-4220-7_15 10.1007/978-1-0716-4220-7_15 39363077

[CR50] Mannazzu I, Domizio P, Carboni G, Zara S, Zara G, Comitini F, Budroni M, Ciani M (2019) Yeast killer toxins: from ecological significance to application. Crit Rev Biotechnol 39(5):603–617. 10.1080/07388551.2019.1601679 31023102 10.1080/07388551.2019.1601679

[CR51] Martinac B, Zhu H, Kubalski A, Zhou XL, Culbertson M, Bussey H, Kung C (1990) Yeast K1 killer toxin forms ion channels in sensitive yeast spheroplasts and in artificial liposomes. Proc Natl Acad Sci USA 87(16):6228-6232. 10.1073/pnas.87.16.6228 10.1073/pnas.87.16.6228 PMC54506 1696721

[CR52] Matuszyńska A, Ebenhöh O, Zurbriggen MD, Ducat DC, Axmann IM (2024) A new era of synthetic biology—microbial community design. Synth Biol 9(1):ysae011. 10.1093/synbio/ysae011 10.1093/synbio/ysae011 PMC11290361 39086602

[CR53] Meškauskas A, Čitavičius D (1992) The K2-type killer toxin- and immunity-encoding region from *Saccharomyces cerevisiae*: structure and expression in yeast. Gene 111(1):135–139. 10.1016/0378-1119(92)90615-V 1547949 10.1016/0378-1119(92)90615-v

[CR54] Miyamoto M, Furuichi Y, Komiyama T (2011) Genome-wide screen of *Saccharomyces cerevisiae* for killer toxin HM-1 resistance. Yeast 28(1):27–41. 10.1002/yea.1818 20803478 10.1002/yea.1818

[CR55] Nishimura K, Kanemaki MT (2014) Rapid depletion of budding yeast proteins via the fusion of an auxin-inducible degron (AID). Curr Protoc Cell Biol 64(1):20.9.1-20.9.16. 10.1002/0471143030.cb2009s64 25181302 10.1002/0471143030.cb2009s64

[CR56] Novotna D, Flegelova H, Janderova B (2004) Different action of killer toxins K1 and K2 on the plasma membrane and the cell wall of. FEMS Yeast Res 4(8):803–813. 10.1016/j.femsyr.2004.04.007 15450187 10.1016/j.femsyr.2004.04.007

[CR57] Oka GU, Souza DP, Sgro GG, Guzzo CR, Dunger G, Farah CS (2024) Xanthomonas immunity proteins protect against the cis-toxic effects of their cognate T4SS effectors. EMBO Rep 25(3):1436–1452. 10.1038/s44319-024-00060-6 38332152 10.1038/s44319-024-00060-6 PMC10933484

[CR58] Pagé N, Gérard-Vincent M, Ménard P, Beaulieu M, Azuma M, Dijkgraaf GJP, Li H, Marcoux J, Nguyen T, Dowse T, Sdicu A-M, Bussey H (2003) A *Saccharomyces cerevisiae* genome-wide mutant screen for altered sensitivity to K1 killer toxin. Genetics 163(3):875–894. 10.1093/genetics/163.3.875 12663529 10.1093/genetics/163.3.875 PMC1462477

[CR59] Paluszynski JP, Klassen R, Meinhardt F (2007) *Pichia acaciae* killer system: genetic analysis of toxin immunity. Appl Environ Microbiol 73(13):Article 13. 10.1128/AEM.00271-07 10.1128/AEM.00271-07 PMC1932769 17483256

[CR60] Pavão G, Sfalcin I, Bonatto D (2023) Biocontainment techniques and applications for yeast biotechnology. Fermentation 9(4):341. 10.3390/fermentation9040341

[CR61] Połomska X, Neuvéglise C, Zyzak J, Żarowska B, Casaregola S, Lazar Z (2021) New cytoplasmic virus-like elements (VLEs) in the yeast *Debaryomyces hansenii*. Toxins 13(9):615. 10.3390/toxins13090615 34564619 10.3390/toxins13090615 PMC8472843

[CR62] Prins RC, Billerbeck S (2025) Small proteins and peptides conferring protection against antimicrobial compounds. Trends Microbiol 33(6):586–602. 10.1016/j.tim.2025.01.011 40055019 10.1016/j.tim.2025.01.011

[CR63] Prins RC, Billerbeck S (2024) The signal peptide of yeast killer toxin K2 confers producer self-protection and allows conversion into a modular toxin-immunity system. Cell Rep 43(7):Article 7. 10.1016/j.celrep.2024.114449 10.1016/j.celrep.2024.114449 38985680

[CR64] Prins RC, Billerbeck S (2025a) Protocol to study toxic, immune, and suicidal phenotypes of killer yeast. STAR Protoc 6(1):103611. 10.1016/j.xpro.2025.103611 39893641 10.1016/j.xpro.2025.103611 PMC11835635

[CR65] Prins RC, Billerbeck S (2025b) Small proteins and peptides conferring protection against antimicrobial compounds. Trends Microbiol 33(6):586–602. 10.1016/j.tim.2025.01.011 40055019 10.1016/j.tim.2025.01.011

[CR66] Prins RC, Marinus T, Dafni E, Yacoby I, Billerbeck S (2026) Alanine scanning of the yeast killer toxin K2 reveals key residues for activity, gain-of-function variants, and supports prediction of precursor processing and 3D structure. J Struct Biol X 13:100142. 10.1016/j.yjsbx.2025.100142 42516764 10.1016/j.yjsbx.2025.100142 PMC13404059

[CR67] Qiu J, Zhai Y, Wei M, Zheng C, Jiao X (2022) Toxin–antitoxin systems: classification, biological roles, and applications. Microbiol Res 264:127159. 10.1016/j.micres.2022.127159 35969944 10.1016/j.micres.2022.127159

[CR68] Ravikumar A, Arzumanyan GA, Obadi MKA, Javanpour AA, Liu CC (2018) Scalable, continuous evolution of genes at mutation rates above genomic error thresholds. Cell 175(7):1946-1957.e13. 10.1016/j.cell.2018.10.021 30415839 10.1016/j.cell.2018.10.021 PMC6343851

[CR69] Riffer F, Eisfeld K, Breinig F, Schmitt MJ (2002) Mutational analysis of K28 preprotoxin processing in the yeast *Saccharomyces cerevisiae*. Microbiology 148(5):1317–1328. 10.1099/00221287-148-5-1317 11988505 10.1099/00221287-148-5-1317

[CR70] Rivero D, Berná L, Stefanini I, Baruffini E, Bergerat A, Csikász‐Nagy A, De Filippo C, Cavalieri D (2015) Hsp12p and PAU genes are involved in ecological interactions between natural yeast strains. Environ Microbiol 17(8):3069–3081. 10.1111/1462-2920.12950 26079802 10.1111/1462-2920.12950

[CR71] Rodríguez-Cousiño N, Gómez P, Esteban R (2017) Variation and distribution of L-A helper totiviruses in *Saccharomyces sensu stricto* yeasts producing different killer toxins. Toxins 9(10):313. 10.3390/toxins9100313 29019944 10.3390/toxins9100313 PMC5666360

[CR72] Rodríguez-Cousiño N, Gómez P, Esteban R (2022) Expression of the K74 killer toxin from *Saccharomyces paradoxus* is modulated by the toxin-encoding M74 double-stranded RNA 5′ untranslated terminal region. Appl Environ Microbiol 88(8):Article 8. 10.1128/AEM.02030-21 10.1128/aem.02030-21 PMC9040610 35389250

[CR73] Rowley PA (2017) The frenemies within: viruses, retrotransposons and plasmids that naturally infect *Saccharomyces* yeasts. Yeast 34(7):279–292. 10.1002/yea.3234 28387035 10.1002/yea.3234

[CR74] Russell PJ, Bennett AM, Love Z, Baggott DM (1997) Cloning, sequencing and expression of a full-length cDNA copy of the M1 double-stranded RNA virus from the yeast, *Saccharomyces cerevisiae*. Yeast 13(9):829–836. 10.1002/(SICI)1097-0061(199707)13:9829::AID-YEA1443.0.CO;2-R 10.1002/(SICI)1097-0061(199707)13:9<829::AID-YEA144>3.0.CO;2-R 9234671

[CR75] Satwika D, Klassen R, Meinhardt F (2012) Anticodon nuclease encoding virus-like elements in yeast. Appl Microbiol Biotechnol 96(2):345–356. 10.1007/s00253-012-4349-9 22899498 10.1007/s00253-012-4349-9

[CR76] Schaffrath R, Meinhardt F, Klassen R (2018) Yeast killer toxins: fundamentals and applications. In: Anke T, Schüffler A (eds) *Physiology and genetics: selected basic and applied aspects*. Springer International Publishing, pp 87–118. 10.1007/978-3-319-71740-1_3

[CR77] Schickel J (1996) *Kluyveromyces lactis* killer system: analysis of cytoplasmic promoters of the linear plasmids. Nucleic Acids Res 24(10):1879–1886. 10.1093/nar/24.10.1879 8657569 10.1093/nar/24.10.1879 PMC145886

[CR78] Schmitt MJ (1995) Cloning and expression of a cDNA copy of the viral K28 killer toxin gene in yeast. Mol Gen Genet 246(2):236–246. 10.1007/BF00294687 7862095 10.1007/BF00294687

[CR79] Stark MJR (1988) Resolution of sequence discrepancies in the ORF1 region of the *Buyveromyces lactis* plasmid k1. Nucleic Acids Res 16(2):771–771. 10.1093/nar/16.2.771 3340559 10.1093/nar/16.2.771 PMC334698

[CR80] Strak MJR, Boyd A, Mileham AJ, Ramonos MA (1990) The plasmid-encoded killer system of *Kluyveromyces lactis*: a review. Yeast 6(1):1–29. 10.1002/yea.320060102 10.1002/yea.320060102 2180235

[CR81] Suzuki Y, Schwartz SL, Mueller NC, Schmitt MJ (2017) Cysteine residues in a yeast viral A/B toxin crucially control host cell killing via pH-triggered disulfide rearrangements. Mol Biol Cell 28(8):1123–1131. 10.1091/mbc.e16-12-0842 28228551 10.1091/mbc.E16-12-0842 PMC5391188

[CR82] Tanguy-Rougeau C, Chen XJ, Wésolowski-Louvel M, Fukuhara H (1990) Expression of a foreign Kmr gene in linear killer DNA plasmids in yeast. Gene 91(1):43–50. 10.1016/0378-1119(90)90160-S 2205539 10.1016/0378-1119(90)90160-s

[CR83] Tokunaga M, Wada N, Hishinuma F (1987a) A novel yeast secretion vector utilizing secretion signal of killer toxin encoded on the yeast linear DNA plasmid pGKL1. Biochem Biophys Res Commun 144(2):613–619. 10.1016/S0006-291X(87)80010-4 3555493 10.1016/s0006-291x(87)80010-4

[CR84] Tokunaga M, Wada N, Hishinuma F (1987b) Expression and identification of immunity determinants on linear DNA killer plasmids pGKL1 and pGKL2 in *Kluyveromyces lactis*. Nucleic Acids Res 15(3):Article 3. 10.1093/nar/15.3.1031 10.1093/nar/15.3.1031 PMC340506 3029695

[CR85] Travers-Cook TJ, Jokela J, Buser CC (2023) The evolutionary ecology of fungal killer phenotypes. Proc R Soc B Biol Sci 290(2005):Article 2005. 10.1098/rspb.2023.1108 10.1098/rspb.2023.1108 PMC10427833 37583325

[CR86] Vadasz AS, Jagganath DB, Pretorius IS, Gupthar AS (2000) Electron microscopy of the K2 killer effect of Saccharomyces cerevisiae T206 on a mesophilic wine yeast. Antonie Van Leeuwenhoek 78:117–122. 10.1023/A:1026588220367 10.1023/a:1026588220367 11204763

[CR87] Valero AM, Lu M, Billerbeck S (2025) Yeast–secreted compounds with antifungal activity – screening, genetic parts, biosynthetic pathways and regulation. FEMS Yeast Res, foaf068. 10.1093/femsyr/foaf068 10.1093/femsyr/foaf068 PMC12715866 41258858

[CR88] Vališ K, Mašek T, Novotná D, Pospišek M, Janderová B (2006) Immunity to killer toxin K1 is connected with the Golgi-to-vacuole protein degradation pathway. Folia Microbiol 51(3):196–202. 10.1007/BF02932122 17004650 10.1007/BF02932122

[CR89] Vijayraghavan S, Kozmin SG, Strope PK, Skelly DA, Magwene PM, Dietrich FS, McCusker JH (2023) RNA viruses, M satellites, chromosomal killer genes, and killer/nonkiller phenotypes in the 100-genomes *S. cerevisiae* strains. G3 (Bethesda) 13(10):jkad167. 10.1093/g3journal/jkad167 37497616 10.1093/g3journal/jkad167 PMC10542562

[CR90] Wemhoff S, Klassen R, Meinhardt F (2014) Site-directed mutagenesis of the heterotrimeric killer toxin zymocin identifies residues required for early steps in toxin action. Appl Environ Microbiol 80(20):6549–6559. 10.1128/AEM.02197-14 25128337 10.1128/AEM.02197-14 PMC4178643

[CR91] Wickner RB, Edskes HK (2015) Yeast killer elements hold their hosts hostage. PLoS Genet 11(5):e1005139. 10.1371/journal.pgen.1005139 25973796 10.1371/journal.pgen.1005139 PMC4431855

[CR92] Yeo C, Abu Bakar F, Chan W, Espinosa M, Harikrishna J (2016) Heterologous expression of toxins from bacterial toxin-antitoxin systems in eukaryotic cells: strategies and applications. Toxins Basel 8(2):49. 10.3390/toxins8020049 26907343 10.3390/toxins8020049 PMC4773802

[CR93] Zhu Y-S-S, Kane J, Zhang X-Y-Y, Zhang M, Tipper DJ (1993) Role of the γ component of preprotoxin in expression of the yeast K1 killer phenotype. Yeast 9(3):Article 3. 10.1002/yea.320090305 10.1002/yea.320090305 8488726

