# Supplementary material for: Extrachromosomally co-encoded toxin-immunity pairs in yeast and their application potential
Source: Appl Microbiol Biotechnol. 2026 May 26;110:234. doi: 10.1007/s00253-026-13876-0 (PMC13442484; doi:10.1007/s00253-026-13876-0)
Supplement: Supplementary file 1 [file 253_2026_13876_MOESM1_ESM.docx]

**Extrachromosomally co-encoded toxin-immunity pairs in yeast and their application potential.
Supplementary material**

Thijs de Vroet^1^, Rianne C. Prins^1^ and Sonja Billerbeck^1,2^

^1^Molecular Microbiology, Groningen Biomolecular Sciences and Biotechnology Institute, University of Groningen, Groningen, 9747 AG, The Netherlands
^2^Department of Bioengineering, Imperial College London, White City Campus Translation & Innovation Hub, London W12 0BZ, UK

*Correspondence: [s.billerbeck@imperial.ac.uk](mailto:s.billerbeck@imperial.ac.uk)

**Supplementary Figure 1.** **Phenotypes of ORFs expressed using (A) nuclear episomal plasmids and the (B) Hybrid VLE system.** Black crosses indicate the absence of the phenotype expected for a given ORF. (A) Expression of the immunity factor ORFs pPACORF4, DrORF5 and KlORF3 on did not result in an immunity phenotype. Expression of A/T decreased versions of these immunity factors PaORF4ms and KlORF3ms resulted in protection against their respective γ-toxin (Kast et al., 2015). Expression of the γ-toxin ORFs PaORF2 (Klassen et al., 2008), DrORF3 (Klassen et al., 2014), PiORF4 (Kast et al., 2014; Klassen & Meinhardt, 2003), and KlORF4 (Wemhoff et al., 2014) all resulted in a cis toxic phenotype. Two of the three hypothetical γ-toxin ORFs (Ht1-3) identified by Henegan et al. also resulted in a cis toxic phenotype(Heneghan et al., 2024). Finally, expression of the α/β chitinase ORF KlORF2 and cysteine substitution variant KlORF2-C250A indicated by * revealed the essential role of this cysteine residue for trans toxicity (Wemhoff et al., 2014). (B) Expression of WT immunity ORFs pPACORF4 (Paluszynski et al., 2007), DrORF5 (Kast et al., 2015), and KlORF3 (Kast et al., 2015) on a hybrid VLE system protects sensitive cells against the cognate toxins.

**Supplementary Figure 2.** Phenotypes of dsRNA toxin chimeras of K28 (Breinig et al., 2006), K2 (Prins & Billerbeck, 2024) and K1 (Gier et al., 2017, 2020). Chimeras consist of various toxin domains, truncated domains, or signal peptides from the Pichia toolkit (αMFΔ and Killer-αMFΔ) (Obst et al., 2017)

## **Supplementary Note 1**

Toxicity is defined by the IUPAC as(Duffus, 1993):

“*Capacity to cause injury to a living organism defined with reference to the quantity of substance administered or absorbed, the way in which the substance is administered (inhalation, ingestion, topical application, injection) and distributed in time (single or repeated doses), the type and severity of injury, the time needed to produce the injury, the nature of the organism(s) affected and other relevant conditions.*“

The assays described in this review have a limited screening range for most of these conditions. For the halo assay and spot assay, the quantity of toxin is limited by the capacity of the toxin producer. This limitation does, in theory, not apply to the microtiter assay as the dose is only limited by solubility; however, in practice addition of toxin is limited by the need for toxin production. Furthermore, all three assays only reveal growth inhibition within several generations of toxin addition, depending on the seeding density of the sensitive strain, thus potentially missing slower killing activity. Additionally, the microtiter plate assay only screens the effect of one-time additions of these toxins. Finally, toxin activity is heavily dependent on pH and temperature. Due to these limitations, extrapolating toxicity phenotypes outside of assay conditions remains challenging. Despite these limitation assays can be adjusted to reflect relevant conditions (e.g., toxins used for biocontainment can be tested in conditions mimicking the inside and outside environment).

## **References**

Breinig, F., Sendzik, T., Eisfeld, K., & Schmitt, M. J. (2006). Dissecting toxin immunity in virus-infected killer yeast uncovers an intrinsic strategy of self-protection. *Proceedings of the National Academy of Sciences of the United States of America*, *103*(10), Article 10. https://doi.org/10.1073/pnas.0510070103

Duffus, J. (1993). *Glossary for chemists of terms used in toxicology (IUPAC Recommendations 1993)*. *Pure and Applied Chemistry*, *65*(9), 2003–2122. https://doi.org/10.1351/pac199365092003

Gier, S., Schmitt, M. J., & Breinig, F. (2017). Expression of K1 toxin derivatives in Saccharomyces cerevisiae mimics treatment with exogenous toxin and provides a useful tool for elucidating K1 mechanisms of action and immunity. *Toxins*, *9*(11), Article 11. https://doi.org/10.3390/toxins9110345

Gier, S., Schmitt, M. J., & Breinig, F. (2020). Analysis of Yeast Killer Toxin K1 Precursor Processing via Site-Directed Mutagenesis: Implications for Toxicity and Immunity. *mSphere*, *5*(1), Article 1. https://doi.org/10.1128/msphere.00979-19

Heneghan, P. G., Salzberg, L. I., & Wolfe, K. H. (2024). *Zymocin-like killer toxin gene clusters in the nuclear genomes of filamentous fungi*. Evolutionary Biology. https://doi.org/10.1101/2024.10.25.620242

Kast, A., Klassen, R., & Meinhardt, F. (2014). rRNA fragmentation induced by a yeast killer toxin. *Molecular Microbiology*, *91*(3), 606–617. https://doi.org/10.1111/mmi.12481

Kast, A., Voges, R., Schroth, M., Schaffrath, R., Klassen, R., & Meinhardt, F. (2015). Autoselection of Cytoplasmic Yeast Virus Like Elements Encoding Toxin/Antitoxin Systems Involves a Nuclear Barrier for Immunity Gene Expression. *PLOS Genetics*, *11*(5), Article 5. https://doi.org/10.1371/JOURNAL.PGEN.1005005

Klassen, R., Kast, A., Wünsche, G., Paluszynski, J. P., Wemhoff, S., & Meinhardt, F. (2014). Immunity factors for two related tRNAGln targeting killer toxins distinguish cognate and non-cognate toxic subunits. *Current Genetics*, *60*(3), Article 3. https://doi.org/10.1007/s00294-014-0426-1

Klassen, R., & Meinhardt, F. (2003). Structural and functional analysis of the killer element pPin1-3 from Pichia inositovora. *Molecular Genetics and Genomics*, *270*(2), Article 2. https://doi.org/10.1007/s00438-003-0920-5

Klassen, R., Paluszynski, J. P., Wemhoff, S., Pfeiffer, A., Fricke, J., & Meinhardt, F. (2008). The primary target of the killer toxin from Pichia acaciae is tRNAGln. *Molecular Microbiology*, *69*(3), 681–697. https://doi.org/10.1111/j.1365-2958.2008.06319.x

Obst, U., Lu, T. K., & Sieber, V. (2017). A Modular Toolkit for Generating Pichia pastoris Secretion Libraries. *ACS Synthetic Biology*, *6*(6), 1016–1025. https://doi.org/10.1021/acssynbio.6b00337

Paluszynski, J. P., Klassen, R., & Meinhardt, F. (2007). Pichia acaciae Killer System: Genetic Analysis of Toxin Immunity. *Applied and Environmental Microbiology*, *73*(13), Article 13. https://doi.org/10.1128/AEM.00271-07

Prins, R. C., & Billerbeck, S. (2024). The signal peptide of yeast killer toxin K2 confers producer self-protection and allows conversion into a modular toxin-immunity system. *Cell Reports*, *43*(7), Article 7. https://doi.org/10.1016/j.celrep.2024.114449

Wemhoff, S., Klassen, R., & Meinhardt, F. (2014). Site-Directed Mutagenesis of the Heterotrimeric Killer Toxin Zymocin Identifies Residues Required for Early Steps in Toxin Action. *Applied and Environmental Microbiology*, *80*(20), 6549–6559. https://doi.org/10.1128/AEM.02197-14
